# Concurrent Topology Optimization of Composite Plates for Minimum Dynamic Compliance

**DOI:** 10.3390/ma15020538

**Published:** 2022-01-11

**Authors:** Heng Zhang, Xiaohong Ding, Weiyu Ni, Yanyu Chen, Xiaopeng Zhang, Hao Li

**Affiliations:** 1 School of Mechanical Engineering, University of Shanghai for Science and Technology, Shanghai 200093, China; zhanghengsh@usst.edu.cn (H.Z.); niweiyu138@sina.com (W.N.); 2 Department of Mechanical Engineering, University of Louisville, Louisville, KY 40292, USA; yanyu.chen@louisville.edu; 3 State Key Laboratory of Structural Analysis for Industrial Equipment, Dalian University of Technology, Dalian 116024, China; zhangxiaopeng@dlut.edu.cn; 4 Department of Mechanical Engineering and Science, Kyoto University, Kyotodaigaku-katsura C3, Nishikyo-ku, Kyoto 615-8540, Japan; li.hao.48z@st.kyoto-u.ac.jp

**Keywords:** concurrent topology optimization, damping composite materials, dynamic compliance, homogenization, composite plates

## Abstract

This paper proposes a novel density-based concurrent topology optimization method to support the two-scale design of composite plates for vibration mitigation. To have exceptional damping performance, dynamic compliance of the composite plate is taken as the objective function. The complex stiffness model is used to describe the material damping and accurately consider the variation of structural response due to the change of damping composite material configurations. The mode superposition method is used to calculate the complex frequency response of the composite plates to reduce the heavy computational burden caused by a large number of sample points in the frequency range during each iteration. Both microstructural configurations and macroscopic distribution are optimized in an integrated manner. At the microscale, the damping layer consists of periodic composites with distinct damping and stiffness. The effective properties of the periodic composites are homogenized and then are fed into the complex frequency response analysis at the macroscale. To implement the concurrent topology optimization at two different scales, the design variables are assigned for both macro- and micro-scales. The adjoint sensitivity analysis is presented to compute the derivatives of dynamic compliance of composite plates with respect to the micro and macro design variables. Several numerical examples with different excitation inputs and boundary conditions are presented to confirm the validity of the proposed methodologies. This paper represents a first step towards designing two-scale composite plates with optional dynamic performance under harmonic loading using an inverse design method.

## 1. Introduction

Thin-walled composite plates have been widely used as structural components in various engineering applications to bear static and dynamic loads. The vibrations caused by the dynamic loads are transmitted to passengers and precision equipment, which reduces the crew’s comfort, affects normal operations, and shortens the service life of high-precision equipment [1]. There is a rising demand to design optimal composites that have a superior combination of high stiffness and exceptional vibration mitigation capability. Over the past few decades, active and passive methods have been developed to improve the dynamic performance of composite structures [2,3,4,5]. Among these methods, incorporating a passive damping material layer into the base plates (i.e., free-layer [6] or constrained-layer [7,8]) is one of the most efficient, robust, and low-cost methods. Intrinsically, the vibration performance of the composite plates is determined by the properties of the damping materials and their topological arrangements on the base plates. Conventional design practices for damping composite architectures are focused on parameter analysis, in which only a few design variables are considered (i.e., the thickness or the size of the damping layer) [9,10]. However, these rely heavily on the designers’ intuition and it is hard to obtain the optimal configurations. These challenges are more notable when optimal microstructural damping configurations and macroscopic arrangements are simultaneously pursued.

Topology optimization (TO) [11,12] is a powerful inverse design technique that does not require predefined shapes. It can be used to generate a free-form optimal configuration that fulfills the functional requirements quantified by the objective functions and constraints. A series of TO methods have been developed to design damping composite structures, which can be broadly classified into two categories: one is to optimize the macrostructure layout of the damping material on the plates [13,14,15,16], while the other is focused on optimizing the composite architectures in microscale [17,18,19,20] using the homogenization method [21,22,23,24]. However, most existing works focus on either the macro- or micro-scale TO. Recent studies show that combining the macrostructure topology optimization with microscale composite material design can significantly improve structural performance. Zhu [25] proposed a concurrent TO strategy to optimize the layout of damping material and the beam size of the lattice core; however, they did not consider the microstructure design problems of the damping layer. Zhang [26] proposed a concurrent TO method to design the free-layer damping structures with a maximum structural modal loss factor. In addition to the damping performance, the vibration response of the structures controlled by dynamic stiffness is equally important. To the authors’ knowledge, so far, limited researches have focused on the multi-scale topology optimization of composite plate structures in a frequency range. In this case, dynamic compliance is often used as the design objective for vibration response design [27,28,29,30,31,32,33]. In these studies, dynamic compliance is calculated using proportional damping, which cannot accurately consider the variation of damping due to the change of damping material configurations.

Inspired by the abovementioned investigations, it can be reasonably expected that developing a multi-scale dynamic optimization model to design the composite plates in a frequency range is likely to obtain a light structure and efficiently suppress structural vibration. The novelties of this paper are as follows:

1. A concurrent TO method that can optimize the dynamic compliance of a two-scale composite plate subjected to harmonic loading in a specified frequency range is developed based on the density method.
2. The complex stiffness model is used to describe the material damping and accurately consider the variation of structural response due to the change of damping material configurations. The mode superposition method is used to calculate the frequency response of the composite plates to reduce the heavy computational burden caused by a large number of sample points in the frequency range during each iteration.
3. In the numerical examples, the effects of the excitation frequency range and positions on the optimal composite plate configurations are investigated. It is found that the damping composite material layout is beneficial to resist the deformation of the eigenmode. Finally, some general design rules for the design of composite structures to improve vibration performance are also summarized.

**Figure 1:**
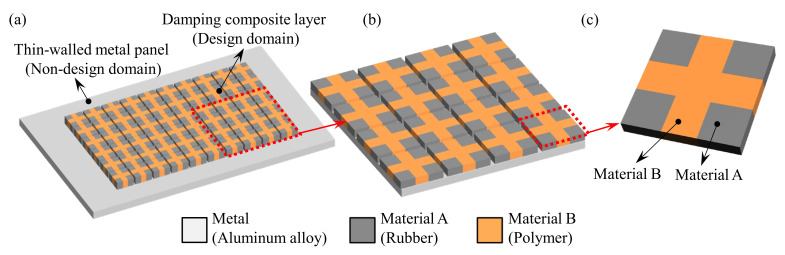
Two-scale composite plate design. (**a**) A composite plate consists of a metal panel and periodic damping composite. (**b**) Periodic damping composite. (**c**) Microstructure of the periodic damping composite.

The composite plate, at the macroscale, consists of a panel as the non-design domain and a damping composite layer as the design domain (see Figure 1a–c). The layout of the optimal damping composite materials on the plates will be optimized. At the microscale, the unit cell of the periodic damping composite is composed of two materials with distinct mechanical properties. One phase is a soft rubber with high damping, while the other is a rigid polymer with high stiffness. The purpose is to improve the structural stiffness and damping simultaneously. To implement the concurrent topology optimization, pseudo density values at the macro and micro-scales are considered as the design variables. The adjoint sensitivity analysis is presented to compute the derivatives of dynamic compliance of composite plates with respect to the two-scale design variables.

The rest of this paper is outlined as follows: Section 2 describes the general numerical computation method of dynamic compliance of the damping composite structures using the finite element method. Section 3 presents the mathematical optimization model of the proposed concurrent topology optimization method and elaborates the sensitivity analysis on the two scales. In Section 4, several numerical examples are presented to demonstrate the effectiveness of the proposed method. Finally, the design rules and conclusions are drawn in Section 5.

## 2. Dynamic Compliance of the Composite Plate

In this section, the dynamic compliance of the proposed composite plate will be derived. For completeness and consistency, details pertaining to the definition of complex stiffness and complex frequency response are provided.

### 2.1. The Complex Stiffness Model for the Damping Material

A complex stiffness model [26] is used to describe the dynamic characteristic of the damping material, which can be stated as:

(1) $$E=E^{\prime }+\zeta E^{\prime \prime }=E^{\prime }\left(1+\zeta \eta \right)$$

where *E′* and *E″* are the storage and loss modulus of the damping material, respectively. *η* is the loss factor of the damping material. *ζ* is the imaginary unit, $\zeta =\sqrt{-1}$.

### 2.2. Complex Frequency Response of the Composite Plate

The momentum equation of a structural system under harmonic excitation can be written as:

(2) $$M\ddot{u}+Ku=fe^{\zeta \omega t}$$

where **M** and **K** are the global mass and stiffness matrices, respectively. **K** is the complex when the structure contains a damping material with a complex stiffness shown in Equation (1). **u** is the displacement vector of the macrostructure, *f* is the magnitude of the harmonic force, *ω* is the excitation frequency of the harmonic force, and *t* is time.

The damping characteristic of the metal panel is ignored in this paper since it is negligibly small compared with that of the damping material. The global stiffness matrix of structure **K** is expressed as:

(3) $$K=K_{p}+K_{v}=K_{p}+K_{v}^{R}+\zeta K_{v}^{I}=K_{}^{R}+\zeta K_{}^{I}$$

where the superscript “R” and “I” represent the real and imaginary parts, respectively. The subscripts “*p*” and “*v*” denote the metal panel and the damping composite layer, respectively.

Considering the free vibration of the composite structure, the complex eigenvalue *λ_r_* and the eigenvector **Φ***_r_* can be expressed as:

(4) $$\lambda_{r}^{2}=\omega_{r}^{2}\left(1+\zeta \eta_{r}\right)$$

(5) $$\Phi_{r}=\Phi_{r}^{R}+\zeta \Phi_{r}^{I}$$

where *ω_r_* and *η_r_* are the *r*-th real eigenvalue and loss factor of the macrostructure. **Φ***_r_*^R^ and **Φ***_r_*^I^ are the real and imaginary parts of the complex eigenvector, respectively. The eigenvector **Φ** is normalized to *ϕ* = {*ϕ*_1_, *ϕ*_2_,…*ϕ*_r_,…}.

Converting the governing equation shown in Equation (2) to the frequency domain leads to:

(6) $$[K^{R}+\zeta K^{I}-\omega^{2}M]ue^{\zeta \omega t}=fe^{\zeta \omega t}$$

The solution of Equation (6) is:

(7) $$u={\left[K^{R}+\zeta K^{I}-\omega^{2}M\right]}^{-1}f=H\left(\omega \right)f$$

Based on the mode superposition method, the response of structure **u** is equal to **H**(*ω*) when the magnitude of the harmonic force is a unit load. Then the response can be described as:

(8) $$H_{jk}\left(\omega \right)=\sum_{r=2}^{\Lambda }\frac{\phi_{rj}\phi_{rk}}{\omega_{r}^{2}-\omega^{2}+\zeta \eta_{r}\omega_{r}^{2}}$$

where *j* is the DoFs of the excitation position, and *k* is the DoFs of the response position. Λ is the number of eigenfrequencies/eigenmodes that are used to calculate the response. Note that the more eigenmodes used, the more accurate results obtained. In this study, Λ = 20 is used.

Using the non-normalized eigenvector **Φ**, Equation (8) can also be expressed as:

(9) $$H_{jk}\left(\omega \right)=\sum_{r=1}^{\Lambda }\frac{\Phi_{rj}\Phi_{rk}}{m_{r}(\omega_{r}^{2}-\omega^{2}+\zeta \eta_{r}\omega_{r}^{2})}$$

where *m_r_* is the *r*-th mode mass.

### 2.3. Dynamic Compliance of the Composite Plate

If the response of the composite plate is obtained, the dynamic compliance can be stated as:

(10) $$C=u^{T}F$$

where **F** is the vector of the applied external load. Due to the complex stiffness matrix, the displacement vector **u** is complex, which is expressed as $u=u_{R}+\zeta u_{I}$. Then the dynamic compliance is given by:

(11) $$C=C_{R}+\zeta C_{I}$$

where *C*_R_ and *C*_I_ are the real and imaginary parts of the dynamic compliance, respectively. The 2-norm of the dynamic compliance of the composite structures can be defined as:

(12) $$d=\left\|C\right\|=\sqrt{C_{R}{}^{2}+C_{I}{}^{2}}$$

According to Equations (6) and (10), compliance can be stated as:

(13) $$C=u^{T}F=u^{T}\left(K^{R}+\zeta K^{I}-\omega^{2}M\right)u$$

The displacement vector is complex, so Equation (13) can be rewritten as:

(14) $$C=u^{T}F=\left(u_{R}^{T}+\zeta u_{I}^{T}\right)\left(K^{R}+iK^{I}-\omega^{2}M\right)\left(u_{R}+\zeta u_{I}\right)$$

Finally, dynamic compliance *C* can be stated as:

(15) $$\begin{array}{l} C=u_{R}^{T}\left(K^{R}-\omega^{2}M\right)u_{R}-u_{R}^{T}\left(K^{I}\right)u_{I}-u_{I}^{T}\left(K^{R}-\omega^{2}M\right)u_{I}-u_{I}^{T}\left(K^{I}\right)u_{R}\\ \;+\zeta \left[u_{R}^{T}\left(K^{R}-\omega^{2}M\right)u_{I}+u_{R}^{T}\left(K^{I}\right)u_{R}+u_{I}^{T}\left(K^{R}-\omega^{2}M\right)u_{R}-u_{I}^{T}\left(K^{I}\right)u_{I}\right] \end{array}$$

According to Equation (15), *C*_R_ and *C*_I_ are expressed as:

(16) $$C_{R}=u_{R}^{T}\left(K^{R}-\omega^{2}M\right)u_{R}-u_{R}^{T}\left(K^{I}\right)u_{I}-u_{I}^{T}\left(K^{R}-\omega^{2}M\right)u_{I}-u_{I}^{T}\left(K^{I}\right)u_{R}$$

(17) $$C_{I}=u_{R}^{T}\left(K^{R}-\omega^{2}M\right)u_{I}+u_{R}^{T}\left(K^{I}\right)u_{R}+u_{I}^{T}\left(K^{R}-\omega^{2}M\right)u_{R}-u_{I}^{T}\left(K^{I}\right)u_{I}$$

## 3. Concurrent Topology Optimization and Sensitivity Analysis

### 3.1. Multi-Scale Modeling Procedure

In the present work, the dynamic compliance for the macrostructure is used as the objective function. First, the homogenization method is used to calculate the effective material properties of the viscoelastic composite, as shown in Figure 1c. Then the effective material properties are used to calculate the stiffness and mass matrices of the composite macrostructure in Equation (2), and the frequency response can be obtained by Equation (8). Finally, Equation (10) is used to calculate the structural dynamic compliance.

### 3.2. Mathematical Model for the Concurrent to Problem

The density-based method is employed to identify the optimal micro and macro configurations of the composite plate. The design variable **X** contains the information at both micro- and macro-scales, **X** = [*χ*^MI^, *χ*^MA^]. If the microstructure of the damping composite material is discretized into *m* elements, the design variable *χ*^MI^ is defined as *χ*^MI^ = [$\chi_{1}^{\mathrm{MI}}$,$\chi_{i}^{\mathrm{MI}}$,... $\chi_{m}^{\mathrm{MI}}$]^T^. Analogously, if the macrostructure of the damping layer is discretized into *n* elements, the design variable *χ*^MA^ is defined as *χ*^MA^ = [$\chi_{1}^{\mathrm{MA}}$,$\chi_{i}^{\mathrm{MA}}$,... $\chi_{m}^{\mathrm{MA}}$]^T^. To maximize the structural dynamic stiffness, minimizing the structural dynamic compliance in the specified frequency range is taken as the design objective. Generally, to avoid numerical problems, the resonant frequency is excluded from the specified excitation frequency range. To design materials with isotropic elastic properties, the isotropy constraint must be implemented in the optimization algorithm. Finally, the mathematical model for the concurrent optimization problem considering the macro- and micro-scales can be formulated as follows:

(18a1)find:X(χiMI,χjMA)(18a2)min:f=d(18a3)s.t.:vMI(χMI)≤fMIv0MI(18a4)vMA(χMA)≤fMAv0MA(18a5)(Re(D11H−D22H))2≤ε(18a6)(Re(D11H+D22H−2(D12H+2D33H)))2≤ε(18a7)(Im(D11H−D22H))2≤ε(18a8)(Im(D11H+D22H−2(D12H+2D33H)))2≤ε(18a9)0<χminMI≤χiMI≤1, i=1,2,…,m(18a10)0<χminMA≤χjMA≤1, j=1,2,…,n

where, **X** is the design variable, which consists of the subsets of design variables for both domains, the micro relative density *χ*^MI^, and the macro relative density *χ*^MA^. In Equation (18a2), the objective is to minimize the dynamic compliance *d* in the excitation frequency range. In Equations (18a3) and (18a4), *f* ^MA^ and *f* ^MI^ are the volume fractions on macro and micro scales, respectively. *v*^MA^ and *v_0_*^MA^ are the maximum allowed volume of the damping composite in the macro scale, while *v*^MI^ and *v_0_*^MI^ are those of the soft material in the micro scale. Isotropy constraints are enforced on both the real and imaginary parts of the elastic matrix. Equations (18a5) and (18a6) are the real part, and Equations (18a7) and (18a8) are the imaginary part. *ε* is the tolerance, which is set to 10^−5^ in this paper. Equations (18a9) and (18a10) define the upper and lower bounds of the design variables *χ*^MI^ and *χ*^MA^, respectively. $\chi_{\min}^{\mathrm{MI}}$ and $\chi_{\min}^{\mathrm{MA}}$ are the small positive values for the lower bound of the design variables to avoid numerical instabilities. *m* and *n* are the total number of elements in the macro and micro models, respectively.

For the plate elements, the elastic stiffness matrix is expressed as:

(19) $$D=\frac{Et^{3}}{12(1-\nu^{2})}\left|\begin{array}{ccc} 1 & \nu & 0\\ \nu & 1 & 0\\ 0 & 0 & \frac{1-\nu }{2} \end{array}\right|$$

where *E* and *μ* are the Young’s modulus and Poisson’s ratio of the material, *t* is the thickness of the plate.

The isotropy conditions can be expressed as:

(20) $$\begin{array}{l} (1)D_{11}=D_{22}\\ (2)D_{11}+D_{22}=D_{12}+D_{21}+4D_{33} \end{array}$$

where *D*_11_, *D*_22_, *D*_12_, *D*_21_, and *D*_33_ are the corresponding elastic tensor shown in Equation (19), *D*_12_ = *D*_21_.

Based on the isotropy conditions expressed as Equation (20), the isotropy constraints shown in Equation (21) can be obtained. Note that, to make the value positive, a square of the function is used.

(21) $$\begin{array}{l} (1){\left(D_{11}-D_{22}\right)}^{2}\;\leq \;\varepsilon \\ (2){\left[D_{11}+D_{22}-\left(D_{12}+D_{21}+4D_{33}\right)\right]}^{2}\;\leq \;\varepsilon \end{array}$$

### 3.3. Sensitivity Analysis

#### 3.3.1. Material Interpolation Scheme

Finite element analyses are conducted concurrently in both macro- and micro-scales. The effective elasticity matrix of the damping composite can be obtained by the homogenization theory. Generally, the element size of the unit cell should be less than the half-wavelength, and the excitation frequency range cannot include the resonant frequency. In this paper, the effect of frequency and temperature on the material damping is not considered; thus the homogenized properties **D**^H^ of the microstructure can be expressed as

(22) $$D^{H}=\frac{1}{\left|Y\right|}\sum_{i=1}^{m}\int_{Y}D^{\mathrm{MI}}\left(I-bu_{i}\right)dY$$

where **u***_i_* is the elemental displacement of the microstructure due to the uniform strain fields.│*Y*│ is the volume of the unit cell. *Y* is the domain of the microstructure. **D**^MI^ is the elastic matrix of the *i*-th element in the microstructure. **I** is an identity matrix.

For the mass matrix, the equivalent physical density *ρ*^H^ of the composite material is:

(23) $$\rho^{H}=\sum_{i=1}^{m}v_{i}\rho_{i}^{\mathrm{MI}}$$

where *v_i_* and *ρ_i_*^H^ are the volume and density of the *i*-th element in the micro-scale, respectively.

To obtain distinct optimal topologies, the solid isotropic material with penalization method (SIMP) [34] is applied to the concurrent topology optimization. The elasticity matrix and density of the *i*-th element in micro-scale can be then written as:

(24) $$D_{i}^{\mathrm{MI}}={\left(\chi_{i}^{\mathrm{MI}}\right)}^{p}D_{1}+\left(1-{\left(\chi_{i}^{\mathrm{MI}}\right)}^{p}\right)D_{2}$$

(25) $$\rho_{i}^{\mathrm{MI}}={\left(\chi_{i}^{\mathrm{MI}}\right)}_{}^{q}\rho_{1}+\left(1-{\left(\chi_{i}^{\mathrm{MI}}\right)}_{}^{q}\right)\rho_{2}$$

where *p* and *q* are the exponents of penalization, here, *p* is 3 and *q* is 1. ***D***_1_ and ***D***_2_ denote the elasticity matrix of materials A and B in the microstructure. *ρ*_1_ and *ρ*_2_ are the density.

Substituting the material interpolation shown in Equation (22) to Equation (24), the effective complex elasticity matrix of the microstructure is:

(26) $$D^{H}=\frac{1}{\left|Y\right|}\sum_{i=1}^{m}\int_{Y}\left({\left(\chi_{i}^{\mathrm{MI}}\right)}^{p}D_{1}+{\left(1-\chi_{i}^{\mathrm{MI}}\right)}^{p}D_{2}\right)\left(I-bu_{i}\right)dY$$

Substituting the material interpolation shown in Equation (23) to Equation (25), the effective density of the microstructure is:

(27) $$\rho^{H}=\sum_{i=1}^{m}\left({\left(\chi_{i}^{\mathrm{MI}}\right)}_{}^{q}\rho_{1}+(1-{\left(\chi_{i}^{\mathrm{MI}}\right)}_{}^{q})\rho_{2}\right)$$

For the macro scale material interpolation, the elasticity matrix of the *j*-th element in the macro scale, ***D****_j_*^MA^, is interpolated as:

(28) $$D_{j}^{\mathrm{MA}}={\left(\chi_{j}^{\mathrm{MA}}\right)}^{p}D^{H}$$

The density of the *j*-th element in the macro model, *ρ_j_*^MA^, is interpolated as:

(29) $$\rho_{j}^{\mathrm{MA}}={\left(\chi_{j}^{\mathrm{MA}}\right)}_{}^{q}\rho^{H}$$

To obtain the structural response of the composite plate, macro-scale finite element analysis should be conducted regarding the microstructural properties. The stiffness and mass matrix of the damping layer **K**^v^ and **M**^v^ are assembled with the elemental stiffness or mass matrix, which can be stated as:

(30) $$K^{v}=\sum_{j}^{n}k_{j}^{v}=\sum_{j}^{n}\int_{\Omega_{j}}B^{T}D^{\mathrm{MA}}Bd\Omega_{j}$$

(31) $$M^{v}=\sum_{j}^{n}m_{j}^{v}=\sum_{j}^{n}\int_{\Omega_{j}}N^{T}\rho^{\mathrm{MA}}Nd\Omega_{j}$$

where **B** and **N** are the strain–displacement matrix and shape function in the macro-scale. **k***_j_*^v^ and **m***_j_*^v^ are the stiffness and mass matrices of the *j*-th element in the macrostructure, respectively. **D**^MA^ is the elastic matrix that can be computed with Equation (28). *ρ*^MA^ is the density that can be computed with Equation (29). Ω is the design domain of the macrostructure.

The sensitivity analysis requires finite element analysis in both macro- and micro-levels. The sensitivity of the macro system’s dynamic compliance due to the change of a design variable on the macro- or micro-scales can be obtained by computing the derivative of *d* given in Equation (12).

#### 3.3.2. Sensitivity Analysis at the Macro-Scale

Considering the change of each element at the macroscale, the sensitivity of the macrostructure design variables *y_i_* can be derived. Using the chain rule, the sensitivity of dynamic compliance expressed in Equation (12) with respect to the design variable *y_j_* can be formulated as:

(32) $$\frac{\partial d}{\partial \chi_{j}^{\mathrm{MA}}}=\frac{1}{d}\left(C_{R}\frac{\partial C_{R}}{\partial \chi_{j}^{\mathrm{MA}}}+C_{I}\frac{\partial C_{I}}{\partial \chi_{j}^{\mathrm{MA}}}\right)$$

According to Equations (16) and (17), the sensitivities of *C*_R_ and *C*_I_ are as follows,

(33) $$\frac{\partial C_{R}}{\partial \chi_{j}^{\mathrm{MA}}}=u_{R}^{T}\left(\frac{\partial K^{R}}{\partial \chi_{j}^{\mathrm{MA}}}-\omega^{2}\frac{\partial M}{\partial \chi_{j}^{\mathrm{MA}}}\right)u_{R}-u_{R}^{T}\left(\frac{\partial K^{I}}{\partial \chi_{j}^{\mathrm{MA}}}\right)u_{I}-u_{I}^{T}\left(\frac{\partial K^{R}}{\partial \chi_{j}^{\mathrm{MA}}}-\omega^{2}\frac{\partial M}{\partial \chi_{j}^{\mathrm{MA}}}\right)u_{I}-u_{I}^{T}\left(\frac{\partial K^{I}}{\partial \chi_{j}^{\mathrm{MA}}}\right)u_{R}$$

(34) $$\frac{\partial C_{I}}{\partial \chi_{j}^{\mathrm{MA}}}=u_{R}^{T}\left(\frac{\partial K^{R}}{\partial \chi_{j}^{\mathrm{MA}}}-\omega^{2}\frac{\partial M}{\partial \chi_{j}^{\mathrm{MA}}}\right)u_{I}+u_{R}^{T}\left(\frac{\partial K^{I}}{\partial \chi_{j}^{\mathrm{MA}}}\right)u_{R}+u_{I}^{T}\left(\frac{\partial K^{R}}{\partial \chi_{j}^{\mathrm{MA}}}-\omega^{2}\frac{\partial M}{\partial \chi_{j}^{\mathrm{MA}}}\right)u_{R}-u_{I}^{T}\left(\frac{\partial K^{I}}{\partial \chi_{j}^{\mathrm{MA}}}\right)u_{I}$$

In which the **K**^R^ and **K**^I^ are stated as follows,

(35) KR=Kp+ReKv

(36) KI=ImKv

The thin-walled metal panel is the non-design domain; thus K^p^ is a constant. Using the material interpolation scheme proposed in Equations (28) and (29), and based on the stiffness ***K***^v^ in Equation (30) and mass matrix **M**^v^ in Equation (31), the sensitivity of $\frac{\partial K^{R}}{\partial \chi_{j}^{\mathrm{MA}}}$, $\frac{\partial K^{I}}{\partial \chi_{j}^{\mathrm{MA}}}$, $\frac{\partial M}{\partial \chi_{j}^{\mathrm{MA}}}$ can be obtained by the following equation.

(37) ∂KR∂χjMA=∂ReKv∂χjMA=∑j=1n∫ΩjBTpχjMAp−1ReDMA∂χiMIBdΩj

(38) ∂KI∂χjMA=∂ImKv∂χjMA=∑j=1n∫ΩjBTpχjMAp−1ImDMA∂χiMIBdΩj

(39) $$\frac{\partial M}{\partial \chi_{j}^{\mathrm{MA}}}=\sum_{j=1}^{n}\int_{\Omega }N^{T}\left(q{\left(\chi_{j}^{\mathrm{MA}}\right)}_{}^{q-1}\rho^{\mathrm{MA}}\right)Nd\Omega_{j}$$

The sensitivity in the macrostructure for maximizing the dynamic compliance of the system is obtained by inserting Equations (37)–(39) to Equations (33) and (34). Then to Equation (32), the sensitivity of the dynamic compliance with respect to the macro design variable *y_j_* can be obtained.

#### 3.3.3. Sensitivity Analysis at the Micro-Scale

Similar to the sensitivity analysis at the macro-scale, the sensitivity of dynamic compliance concerning design variable *x_i_* in micro-scale can be obtained as:

(40) $$\frac{\partial d}{\partial \chi_{i}^{\mathrm{MI}}}=\frac{1}{d}\left(C_{R}\frac{\partial C_{R}}{\partial \chi_{i}^{\mathrm{MI}}}+C_{I}\frac{\partial C_{I}}{\partial \chi_{i}^{\mathrm{MI}}}\right)$$

where

(41) $$\frac{\partial C_{R}}{\partial \chi_{i}^{\mathrm{MI}}}=u_{R}^{T}\left(\frac{\partial K^{R}}{\partial \chi_{i}^{\mathrm{MI}}}-\omega^{2}\frac{\partial M}{\partial \chi_{i}^{\mathrm{MI}}}\right)u_{R}-u_{R}^{T}\left(\frac{\partial K^{I}}{\partial \chi_{i}^{\mathrm{MI}}}\right)u_{I}-u_{I}^{T}\left(\frac{\partial K^{R}}{\partial \chi_{i}^{\mathrm{MI}}}-\omega^{2}\frac{\partial M}{\partial \chi_{i}^{\mathrm{MI}}}\right)u_{I}-u_{I}^{T}\left(\frac{\partial K^{I}}{\partial \chi_{i}^{\mathrm{MI}}}\right)u_{R}$$

(42) $$\frac{\partial C_{I}}{\partial \chi_{i}^{\mathrm{MI}}}=u_{R}^{T}\left(\frac{\partial K^{R}}{\partial \chi_{i}^{\mathrm{MI}}}-\omega^{2}\frac{\partial M}{\partial \chi_{i}^{\mathrm{MI}}}\right)u_{I}+u_{R}^{T}\left(\frac{\partial K^{I}}{\partial \chi_{i}^{\mathrm{MI}}}\right)u_{R}+u_{I}^{T}\left(\frac{\partial K^{R}}{\partial \chi_{i}^{\mathrm{MI}}}-\omega^{2}\frac{\partial M}{\partial \chi_{i}^{\mathrm{MI}}}\right)u_{R}-u_{I}^{T}\left(\frac{\partial K^{I}}{\partial \chi_{i}^{\mathrm{MI}}}\right)u_{I}$$

where the sensitivity of ***K***^R^ with respect to the micro-scale design variable *x_i_* is formulated as:

(43) ∂KR∂χiMI=∑j=1n∫ΩjBT∂χjMApReDMA∂χiMIBdΩj  =∑j=1nχjMAp∫ΩjBT1Y∫YI−buTp(χiMI)p−1ReD1+p(1−χiMI)p−1ReD2I−budYBdΩ

The sensitivity of ***K***^I^ with respect to the micro-scale design variable *x_i_* is formulated as:

(44) ∂KI∂χiMI=∑j=1n∫ΩjBT∂ρjMApImDMA∂χiMIBdΩj  =∑j=1nχjMAp∫ΩjBT1Y∫YI−buTp(χiMI)p−1ImD1+p(1−χiMI)p−1ImD2I−budYBdΩ

The sensitivity of the mass matrix with respect to the micro-scale design variable *x_i_* is:

(45) $$\frac{\partial M}{\partial \chi_{i}^{\mathrm{MI}}}=\sum_{j=1}^{n}\int_{\Omega }N^{T}\frac{\partial \left({\left(\chi_{j}^{\mathrm{MA}}\right)}_{}^{q}\rho^{\mathrm{MA}}\right)}{\partial \chi_{i}^{\mathrm{MI}}}Nd\Omega_{j}=\sum_{j=1}^{n}{\left(\chi_{j}^{\mathrm{MA}}\right)}_{}^{q}\int_{\Omega }N^{T}\left[q{\left(\chi_{i}^{\mathrm{MI}}\right)}_{}^{q-1}\left(\rho_{1}-\rho_{2}\right)\right]\;Nd\Omega_{j}$$

Substituting Equations (43)–(45) into Equations (41) and (42), and then into Equation (40), the sensitivity of the dynamic compliance with respect to the micro design variable *x_i_* can be obtained.

### 3.4. Design Process

**Figure 2:**
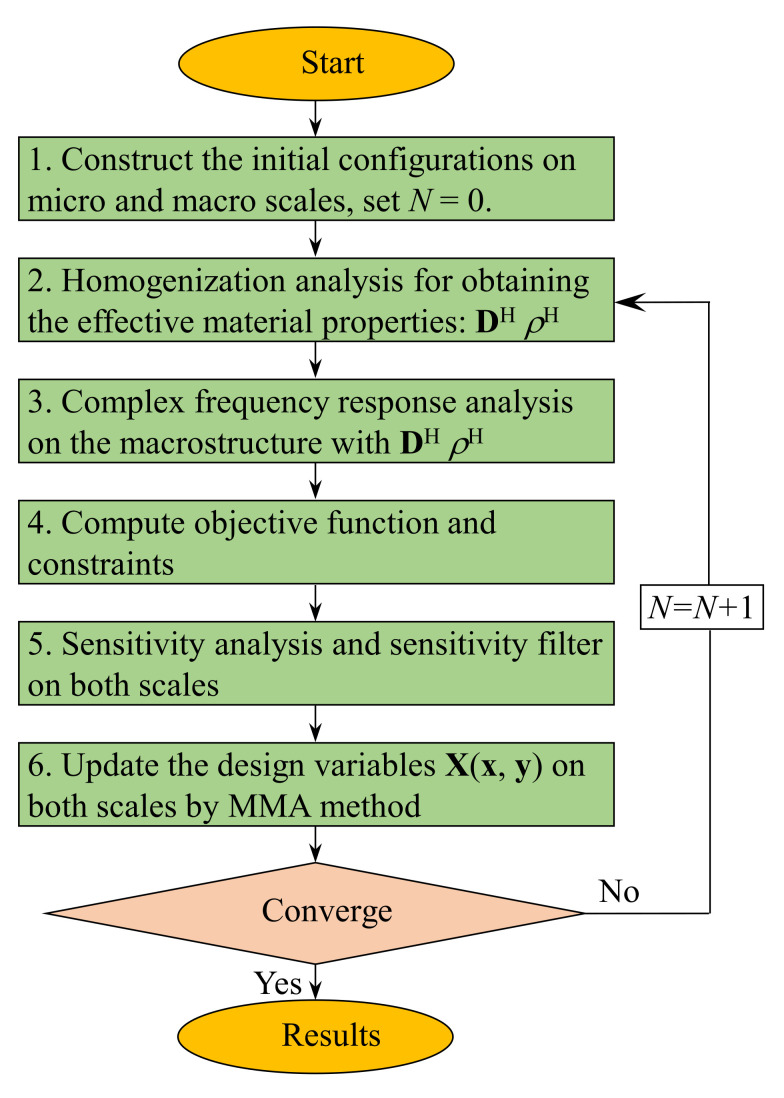
Flowchart of the concurrent topology optimization algorithm.

The mathematical optimization problem expressed in Equation (18) is solved by the method of moving asymptotes (MMA) [35]. The flow chart of the proposed method is illustrated in Figure 2, which includes the following steps:

1. Construct the initial configurations in micro- and macro-scales. Set the initial guess for design variables *x* and *y*. In this study, the initial value of the iteration number is *N* = 0;
2. Calculate the effective properties of the damping composite material. The homogenization method is used to calculate the effective material properties **D**^H^ and *ρ*^H^;
3. Perform the finite element analysis on a macro-scale, using **D**^H^ and *ρ*^H^ to calculate the frequency response of the macrostructure;
4. Compute the objective and constraints;
5. Analyze the sensitivity of the design variables in both macro-and micro-scales and apply the sensitivity filter to avoid checkboard patterns or gray-scale elements;
6. Update the design variables **X**(**x**,**y**) by the MMA method;
7. Repeat steps 2–6 until the objective function is convergent or the iteration number *N* reaches the predefined maximum value *N*_max_ (in this paper, *N*_max_ = 200).

## 4. Model Verification and Numerical Examples

### 4.1. Model Verification of Frequency Response of Composite Plates

In this section, we verify the proposed multi-scale model for composite plates by comparing the results from the full-scale model with those from the commercial finite element tool. The density, Young’s modulus, Poisson’s ratio, and loss factor of the base materials used in all simulations are listed in Table 1.

**Figure 3:**
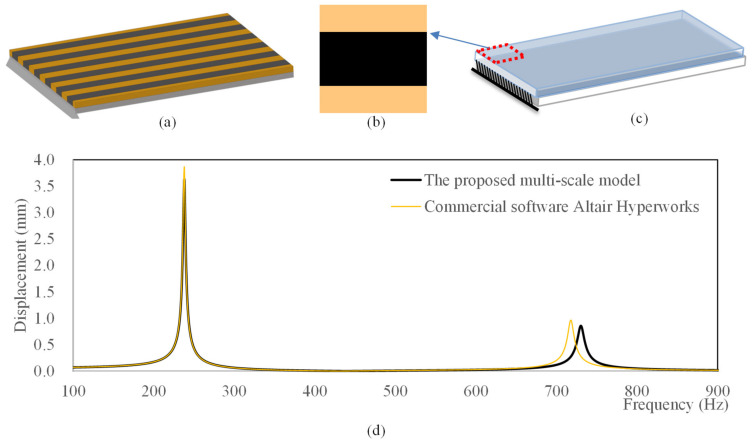
Comparison of the proposed model and commercial finite element tool Altair Hyperworks. (**a**) Full-scale model in Altair Hyperworks. (**b**) Microstructure. (**c**) The proposed multi-scale model. (**d**) Comparison of the displacement frequency response.

Several cantilever structures with different damping layers are modeled using the proposed model and commercial finite element tool Altair Hyperworks. The dimension of the plate is 80 × 60 mm, and the thickness of each layer is 2 mm. Three types of structures are considered: the first is a plate fully covered with polymer, the second is a plate fully covered with rubber, and the third is a plate full of microstructure shown in Figure 3b. In the commercial tool, the full-scale model is established with 320 × 240 elements, which is a single-scale analysis. In the proposed model, the elements of macrostructure are 8 × 6, and the elements of microstructure are 40 × 40. The comparison results are shown in Table 2. It can be found that both the modal loss factor and eigenfrequency have minor errors. The errors of the eigenfrequency are no more than 1.82%, and the errors of the modal loss factor are no more than 8.38%. This demonstrated the computational accuracy of the proposed multi-scale model. From the CPU time, it can be found that the multi-scale model can dramatically decrease the computational cost. The displacement frequency responses of the structures shown in Figure 3a,c are shown in Figure 3d. From the comparison result, it can be found that the displacement of the proposed multi-scale model is nearly the same as the result of commercial software, which demonstrates the accuracy of the proposed multi-scale model.

### 4.2. Numerical Examples

**Figure 4:**
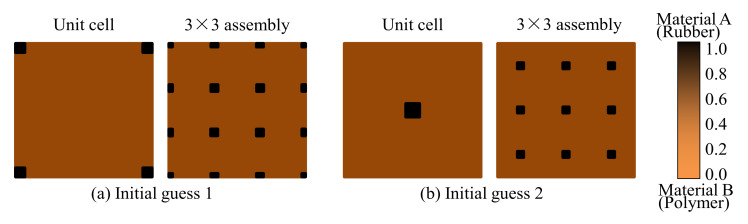
Initial microstructures for topology optimization. (**a**) Initial guess 1 and (**b**) Initial guess 2.

Having demonstrated the accuracy of the proposed model, we will present several numerical examples to demonstrate the effectiveness of the proposed concurrent topology optimization method in the rest of this paper. A composite plate composed of a thin-walled metal panel and damping composite is chosen as the model system. In all the examples, both thicknesses of the thin-walled metal panel and damping material layer are 2 mm. The plate structures with different sizes and boundary conditions are studied. Three examples will be demonstrated, including (1) a rectangular cantilever plate, (2) a rectangular plate clamped with two opposite edges, and (3) a rectangular plate fixed at the four corner points. In all the examples, different excitation frequencies at different excitation positions are studied. For macro-scale TO, a uniform density field is often used as the initial guess for the gradient-based MMA optimization algorithm. However, in micro-scale design, this may lead to a halt of the optimization procedure, this is because a uniformly distributed density field often leads to a uniformly distributed sensitivity field under the periodic boundary condition assumption [26,36]. In this paper, two different non-uniform density fields are used as the initial guesses, as shown in Figure 4. Figure 4a show the initial guess 1 with the density of four corners set to be 1.0, and Figure 4b show the initial guess 2 with the density of the center point set to be 1.0. The other parts of the two initial guesses are set to a uniform distribution with the given volume fraction. From the 3 × 3 assembly of the initial guess microstructure, the two initial guesses are essentially the same. The structures in both scales are discretized using Mindlin plate elements. For simplicity, the macrostructure is discretized using the square element with a size of 5 × 5 mm. The microstructure meshes feature 40 × 40 elements. A filter radius of two times the element size is used in macro- and micro-scales.

#### 4.2.1. Cantilever Composite Plate

**Figure 5:**
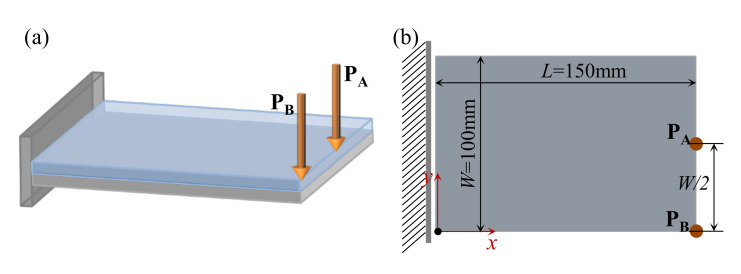
A cantilever composite plate. (**a**) 3D view. (**b**) Top view of the composite plate.

**Figure 6:**
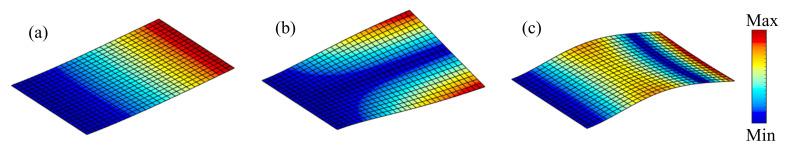
The first three eigenmodes of the cantilever composite plate. (**a**) The first eigenmode at 75 Hz. (**b**) The second eigenmode at 255 Hz. (**c**) The third eigenmode at 467 Hz.

Here we consider the concurrent topology optimization of a rectangular cantilever plate, as illustrated in Figure 5. The dimension of the plate is 150 × 100 × 2 mm. To obtain the vibration response of the plate, frequency response analysis is conducted, in which a harmonically concentrated unit load is applied. Two different excitation locations, P_A_ (*L*, *W*/2) and P_B_ (*L*, 0), are considered. Figure 6 shows the first three eigenmodes with corresponding eigenfrequencies of 75, 255, and 467 Hz. The first eigenmode is bending, the second eigenmode is torsion, and the third eigenmode is second-order bending.

**Figure 7:**
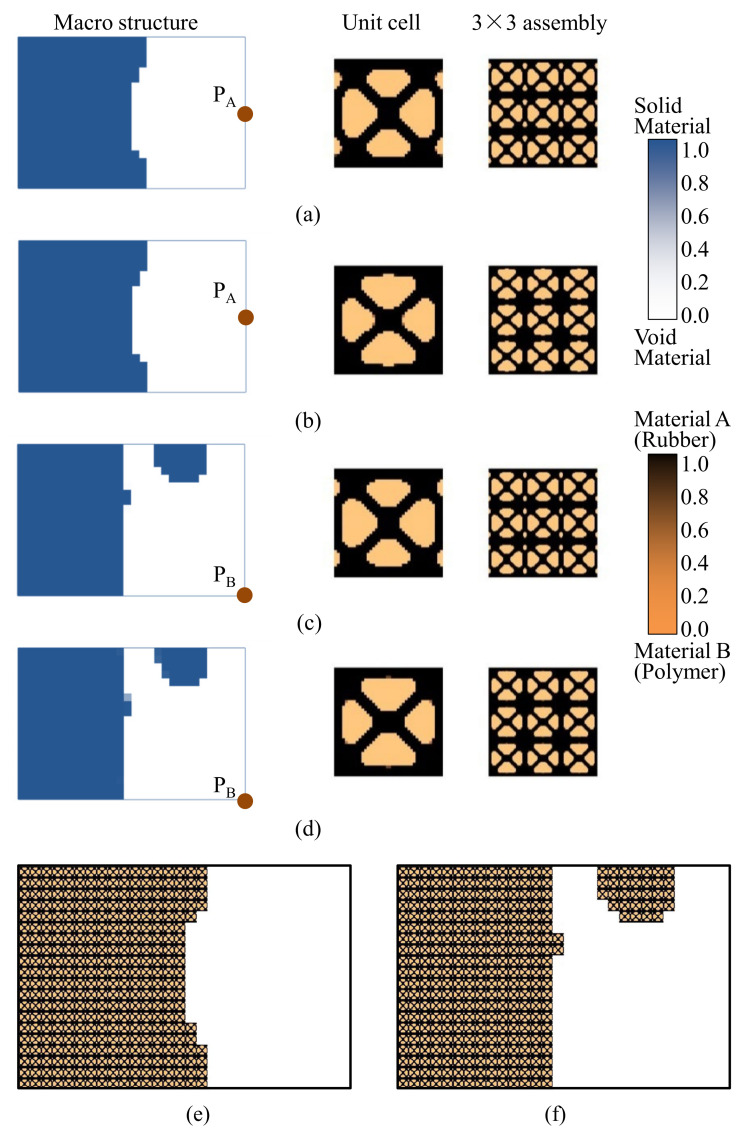
Optimal composite plates under different initial microstructures and excitation positions. The excitation frequency range is 0–60 Hz. (**a**) Initial guess 1, excitation position P_A_, the dynamic compliance of the optimal structure is 5.540 N∙mm. (**b**) Initial guess 2, excitation position P_A_, the dynamic compliance of the optimal structure is 5.580 N∙mm. (**c**) Initial guess 1, excitation position P_B_, the dynamic compliance of the optimal structure is 6.720 N∙mm. (**d**) Initial guess 2, excitation position P_B_, the dynamic compliance of the optimal structure is 6.784 N∙mm. (**e**,**f**) are the overall composite plates for the results shown in (**a**,**c**).

**Figure 8:**
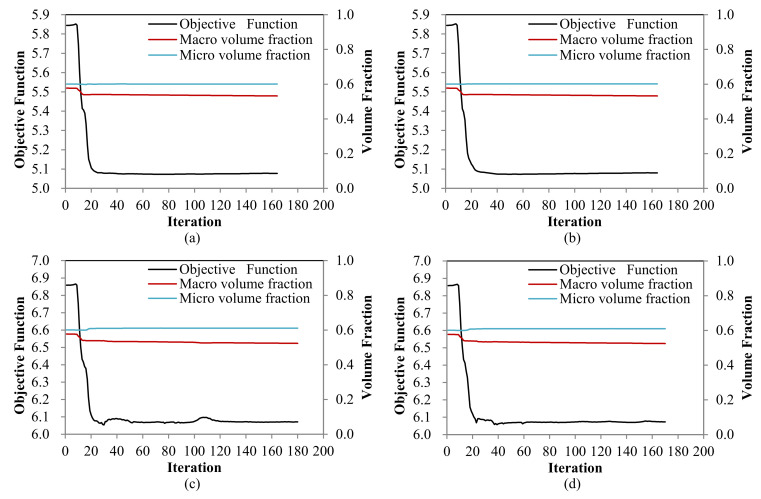
Iteration histories. (**a**–**d**) are the iteration histories of design cases shown in Figure 7a–d, respectively.

First, the effects of the different initial microstructures and excitation positions are studied. The excitation frequency range is set to 0~60 Hz. Four different cases are considered, including (a) the excitation position is at P_A_ and the initial guess 1 is used for the microstructure design, (b) the excitation position is at P_A_ and initial guess 2, (c) the excitation position is at P_B_ and initial guess 1, and (d) the excitation position is at P_B_ and initial guess 2. In all the four test cases, the volume fractions in both scales are the same, which are *f* ^MA^ = 0.5 and *f* ^MI^ = 0.6. When *f* ^MI^ = 0.6, the volume fraction of the damping material A (rubber) is *v*^MI,1^ = 60%. Note that *v*^MI,1^+ *v*^MI,2^ = 1.0, thus the volume fraction of the damping material B (polymer) is *v*^MI,2^ = 40%. The optimal solutions are shown in Figure 7, in which, from left to right, is the damping composite layout in macro-scale, one unit cell of the damping composite in micro-scale, and 3 × 3 assembly of the unit cell. In the macro-scale, the blue domain is the damping composite material, and the white represents the void. On the micro-scale, the black and the copper color represent the rubber and the polymer, respectively. Figure 8 shows the iterative histories of the design cases (a)–(d), respectively. Based on the iteration histories, it can be found that all the solutions are convergent.

When the exciting position is at P_A_, the optimal material configurations are shown in Figure 7a,b. Interestingly, the optimal macrostructures are the same. The damping composite material is mainly located at the left half part of the plate. This is because the excitation frequency range is set to 0–60 Hz, which is below the first eigenfrequency of the base plate 75 Hz. As a result, this material layout is useful to resist the first bending eigenmode. The microstructure of the composite material is similar. The corresponding dynamic compliances of the optimal results shown in Figure 7a,b are 5.540 N∙mm and 5.580 N∙mm, respectively. When the exciting position is at P_B_, the optimal material configurations are shown in Figure 7c,d. Likewise, the optimal macrostructures are the same. The microstructure is the same as the results at excitation point P_A_. The dynamic compliances of Figure 7c,d are 6.720 N∙mm and 6.784 N∙mm, respectively. These results imply that the proposed methodology can mitigate the dependency on the initial guess. When the excitation positions are different, the microstructures are similar, but the macrostructures are different. The optimal macrostructures are obtained to resist the first eigenmode. To show the optimized overall multi-scale composite structures, the result in Figure 7a is shown in Figure 7e, and the result in Figure 7c is shown in Figure 7f.

**Figure 9:**
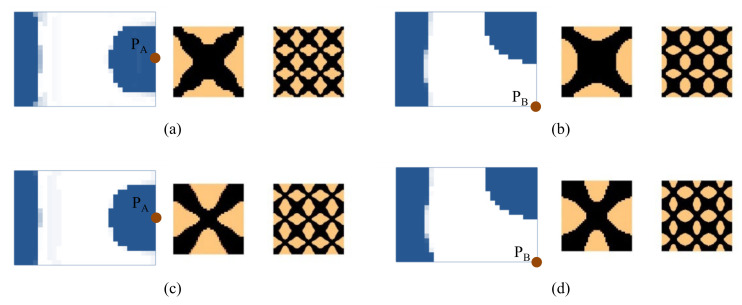
Design results with different initial guesses and exciting positions. The excitation frequency range is 90–200 Hz. (**a**) Initial guess 1, excitation position P_A_. Dynamic compliance 1.720 N∙mm. (**b**) Initial guess 1, excitation position P_B_. Dynamic compliance 3.207 N∙mm. (**c**) Initial guess 2, excitation position P_A_. Dynamic compliance 1.724 N∙mm. (**d**) Initial guess 2, excitation position P_B_. Dynamic compliance 3.211 N∙mm.

**Figure 10:**
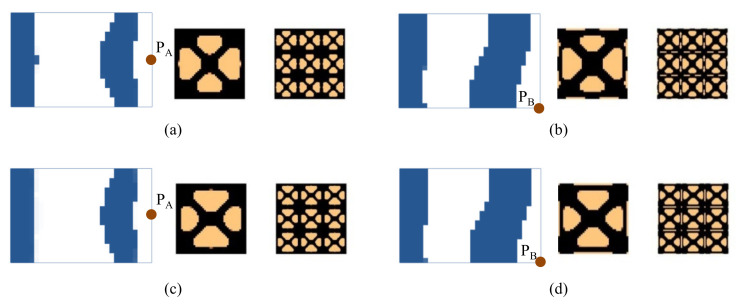
Design results with different initial guesses and excitation positions. The excitation frequency range is 300–400 Hz. (**a**) Initial guess 1, excitation position P_A_. Dynamic compliance 4.757 N∙mm. (**b**) Initial guess 1, excitation position P_B_. Dynamic compliance 6.755 N∙mm. (**c**) Initial guess 2, excitation position P_A_. Dynamic compliance 4.751 N∙mm. (**d**) Initial guess 2, excitation position P_B_. Dynamic compliance 6.732 N∙mm.

Next, we studied the effect of the excitation frequency range on the optimal solutions. Two design examples with the frequency ranges of 90–200 Hz (between the first and second eigenfrequency of the base plate, which is 75 Hz and 255 Hz) and 300–400 Hz (between the second and third eigenfrequency of the base plate, which is 255 Hz and 467 Hz) are studied. The design results for the case where the excitation frequency range is 90–200 Hz are shown in Figure 9, and for the case of 300–400 Hz are shown in Figure 10. Compared with the results of 0–60 Hz shown in Figure 7, the optimal material configurations in both macro- and micro-scales vary with the excitation frequency. For those test cases where the excitation position is placed at P_A_ when the frequency range is 100–200 Hz, the damping material is located at the left side, and they are concentrated around the excitation position to resist the second torsion eigenmode. The composite microstructures and the dynamic compliance of the optimal multi-scale structure obtained by the different initial guesses 1 and 2 are the same, as shown in Figure 9a,c. When the frequency range is 300–400 Hz, the damping material is located at both left and right sides of the plate to resist the third eigenmode, which is second-order bending. The resultant microstructures are shown in Figure 10a,c. For those test cases where the excitation point is placed at P_B_, when the frequency range is 90–200 Hz, the results are shown in Figure 9b,d. The damping material is mainly distributed at the left half and upper right corner of the plate. This is quite different from the solutions obtained when the excitation point is placed at P_A_. This material configuration can resist the torsion eigenmode. The microstructure is similar to the ones shown in Figure 9a,c. When the frequency range is 300–400 Hz, the results are depicted in Figure 10b,d. Similar conclusions can be obtained. From Figure 7, Figure 9 and Figure 10, we can find that when the excitation position is different, the material layouts in both scales are different. This is because the response at position *k* corresponds to the excitation position *j* and excitation frequency *ω*, as given in Equation (9), which means that if the excitation position changes, the response of the whole structure will be changed, which in turn, leads to different material layouts.

Table 3 summarizes the dynamic stiffness and mass comparison of the different results. The mass ratio of the damping layer is defined as the ratio of the mass of the damping layer to the total mass of the base plate. Compared with the base panel, the structures which are fully covered with rubber or polymer have higher mass increasing rates, which are 55.56% and 37.06%, respectively. When covered with rubber, the structural eigenfrequency decreases. When covered with polymer, the structural eigenfrequency is close to that of the base plate. Compared with the base plate, the multi-scale optimized results have a lower mass ratio, and the average mass ratio is approximately 22%. The dynamic stiffness has a minor decrease, and the maximum decrease is 9%. For all the six design results in the table, the average eigenfrequency decreasing rate at the first, second, and third eigenfrequencies are 5.9%, 5.8%, and 4.7%, respectively. For the optimal structures, the damping material is distributed in the most efficient position for vibration reduction. High stiffness, high damping, and lightweight composite structures are obtained.

**Figure 11:**
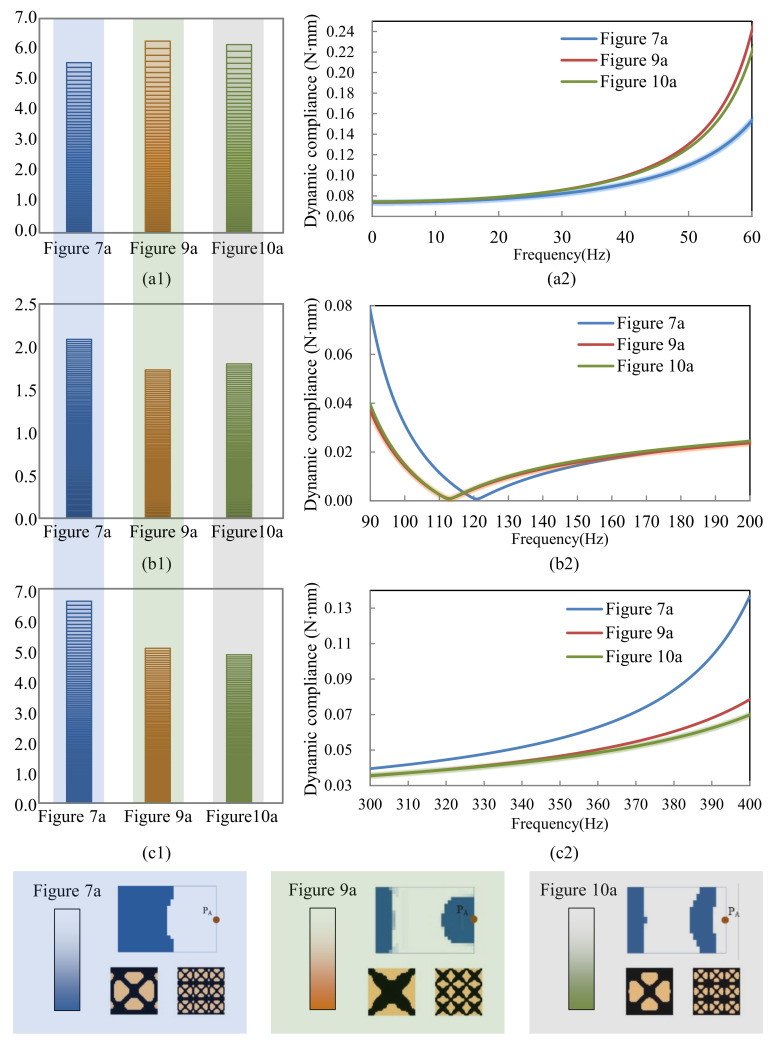
Dynamic compliance of the results shown in Figure 7a, Figure 9a and Figure 10a when thr excitation point is at P_A_. (**a1**) the sum of dynamic compliance 0–60 Hz, (**a2**) dynamic compliances vary with frequency 0–60 Hz. (**b1**) the sum of dynamic compliance 90–200 Hz, (**b2**) dynamic compliances vary with frequency 90–200 Hz. (**c1**) the sum of dynamic compliance 300–400 Hz, (**c2**) dynamic compliances vary with frequency 300–400 Hz.

**Figure 12:**
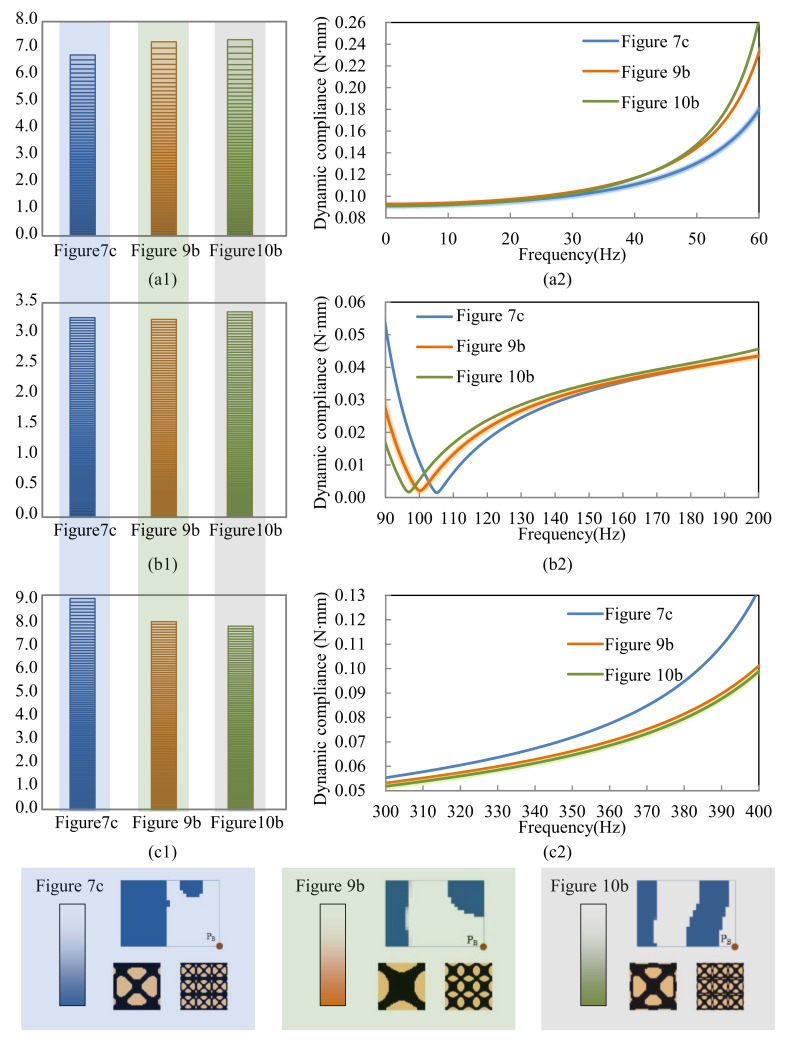
Dynamic compliance of the results is shown in Figure 7c, Figure 9b and Figure 10b when the excitation point is at P_B_. (**a1**) the sum of dynamic compliance 0–60 Hz, (**a2**) dynamic compliance in the frequency range 0–60 Hz. (**b1**) the sum of dynamic compliance 90–200 Hz, (**b2**) dynamic compliance in the frequency range 90–200 Hz. (**c1**) the sum of dynamic compliance 300–400 Hz, (**c2**) dynamic compliance in the frequency range 300–400 Hz.

Figure 11 shows the dynamic compliance of the optimal multi-scale structures when the excitation position is placed at P_A_, as shown in Figure 7a, Figure 9a and Figure 10a. Figure 11(a2) show the dynamic compliance from 0 Hz to 60 Hz, and Figure 11(a1) show the sum of dynamic compliance from 0 Hz to 60 Hz. The optimal structure in Figure 7a has the minimum dynamic compliance in the frequency range of 0–60 Hz. This is because the result of Figure 7a is obtained to minimize the dynamic compliance from 0 Hz to 60 Hz. Figure 11(b2) show the dynamic compliance from 90 Hz to 200 Hz, Figure 11(b1) is the sum of dynamic compliance from 90 Hz to 200 Hz. The figures show that the result of Figure 9a, which is obtained to minimize the dynamic compliance from 90 Hz to 200 Hz, has the minimum dynamic compliance in the frequency range of 90–200 Hz. Figure 11(c1,c2) are the dynamic compliances at 300–400 Hz; the design result of Figure 12a has the minimum dynamic compliance in the frequency range of 300–400 Hz. A similar conclusion can be drawn from Figure 12. They can be summarized as follows: the optimal multi-scale structure obtained with the objective of minimizing the dynamic compliance from *ω*_min_ to *ω*_max_ (i.e., 0–60 Hz, 90–200 Hz, or 300–400 Hz in this paper) has the minimum dynamic compliance in the same frequency range *ω*_min_~*ω*_max_. This demonstrates the effectiveness of the proposed method.

For all microstructures, though different initial guesses are used, we can obtain similar optimal solutions. This further demonstrates that the proposed concurrent topology method is robust for obtaining an optimal solution with different initial guess microstructures. In the rest of this paper, we will use only the initial guess 1.

**Table 3:** Dynamic stiffness and mass comparison of different results.

Examples	Natural Frequency (Hz)			Total Mass (g)	Mass of Damping Layer (g)	Mass Ratio of the Damping Layer
	First	Second	Third			
Base plate	75.1	254.7	467.4	81.00	0.00	0.00%
Fully covered with Rubber	60.5	205.1	376.6	126.00	45.00	55.56%
Fully covered with Polymer	75.7	253.1	470.1	111.02	30.02	37.06%
Figure 7a	75.3	248.9	425.3	101.76	20.76	25.63%
Figure 7c	74.3	244.4	432.8	101.43	20.43	25.22%
Figure 9a	68.1	244.3	450.7	95.75	14.75	18.21%
Figure 9b	69.6	238.6	450.9	96.36	15.36	18.96%
Figure 10a	69.0	235.4	462.8	96.17	15.17	18.73%
Figure 10b	67.7	228.7	450.0	101.08	20.08	24.79%

**Figure 13:**
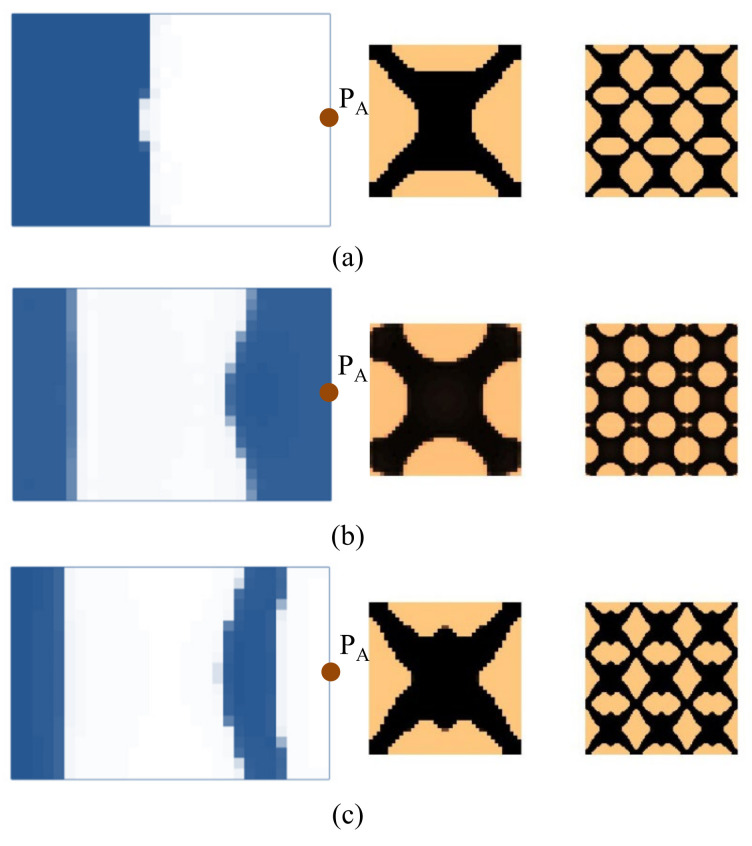
Comparison of three different designs by varying the excitation frequency. (**a**) Excitation frequency 60 Hz. (**b**) Excitation frequency 200 Hz. (**c**) Excitation frequency 400 Hz.

**Figure 14:**
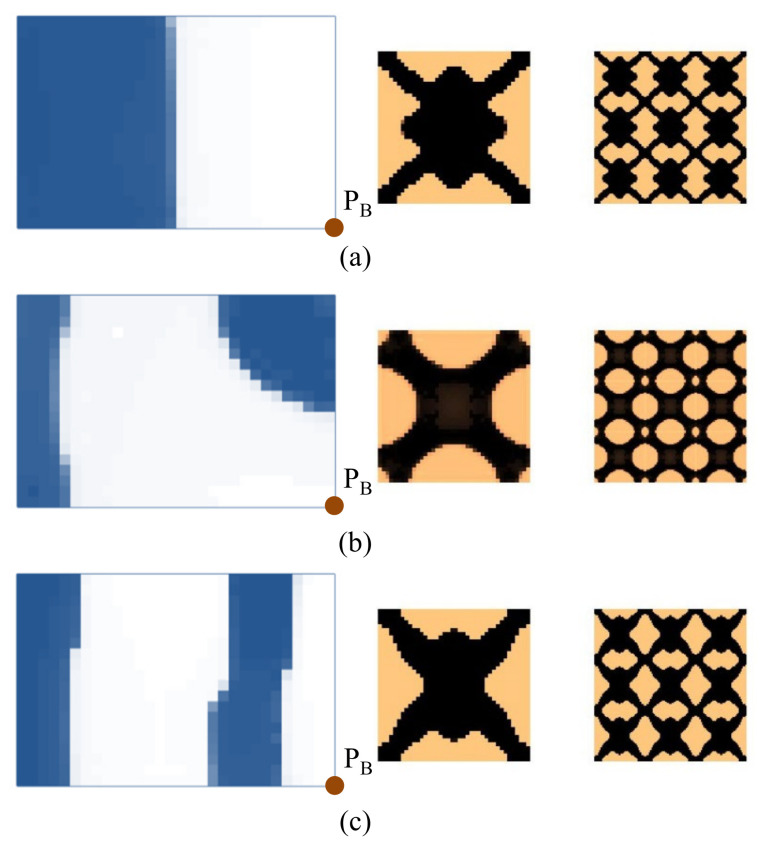
Comparison of three different designs by varying the excitation frequency. (**a**) Excitation frequency 60 Hz. (**b**) Excitation frequency 200 Hz. (**c**) Excitation frequency 400 Hz.

To further demonstrate the effectiveness of the proposed optimization method, the excitation frequencies of the external force at fixed frequencies are discussed. Three test cases under different excitation frequencies are studied hereinafter. The excitation frequencies are set to 60 Hz, 200 Hz, and 400 Hz, which are lower than the first, second, and third eigenfrequencies of the base panel. Figure 13 shows the design results for the case where the excitation position is placed at P_A,_ and Figure 14 shows the results for the case of P_B_. It can be found that with the changes in the excitation frequency, the macro and microstructures are different. From the macrostructure results, when the excitation frequency is 60 Hz, similar material topologies are obtained as the excitation frequency ranges 0–60 Hz. Similar conclusions can be drawn when the excitation frequencies are set to 200 Hz or 400 Hz. Distinct topologies are obtained in both scales for all the test cases.

From all results, we can find that the optimized topologies in microscale look similar to “X” or “☒”. This is mainly because of the isotropic constraint. The “X” or “☒” types are the basic shapes that have isotropic characteristics. Though some similar topologies have been obtained, the optimized microstructures have some minor differences in detail. From the sensitivity analysis Equations (32) and (40), we notice that the sensitivity corresponds to the structural response; different excitation frequencies can lead to different structural responses and lead to minor differences. 

The proposed methodology, in which the excitation frequency can be set according to the design requirement, can facilitate the development of practical solutions that meet engineering requirements to effectively reduce vibration levels in real-world engineering structures.

#### 4.2.2. Composite Plate Clamped with Two Opposite Edges

**Figure 15:**
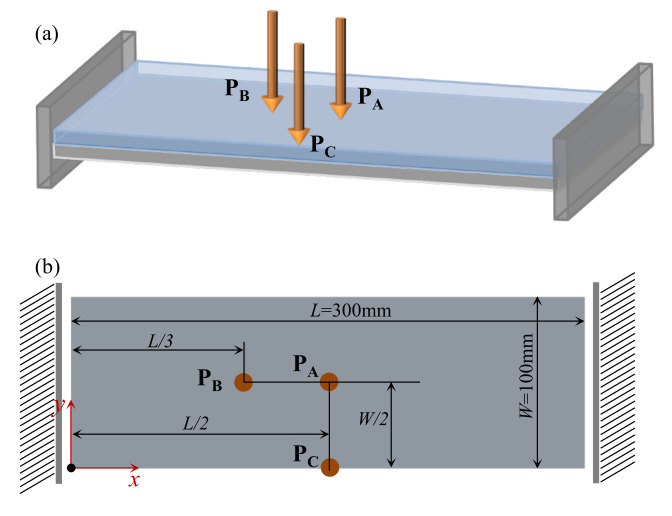
A composite plate clamped on two opposite edges. (**a**) Three-dimensional view. (**b**) Top view of the composite plate.

**Figure 16:**
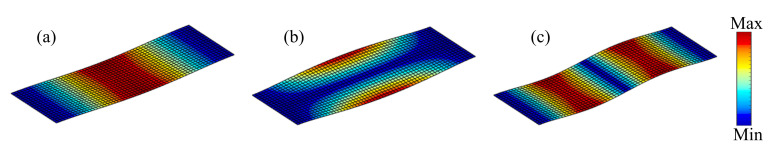
The first three eigenmodes of the plate clamped on two opposite edges. (**a**) The first eigenmode at 122 Hz. (**b**) The second eigenmode at 263 Hz. (**c**) The third eigenmode at 336 Hz.

Figure 15 shows a rectangular plate that is fixed at two opposite sides. The dimension of the plate is *L* = 300 mm, *W* = 100 mm. Three different loading conditions are considered in this example: (1) the excitation position is located at P_A_ (*L*/2, *W*/2), (2) the excitation point is located at P_B_ (*L*/3, *W*/2), and (3) the excitation point is located at P_C_ (*L*/2, 0). The excitation frequency ranges of all three cases are 50–100 Hz. Figure 16 shows the first three eigenmodes associated with eigenfrequencies of 122, 263, and 336 Hz, which are bending, torsion, and second-order bending, respectively.

**Figure 17:**
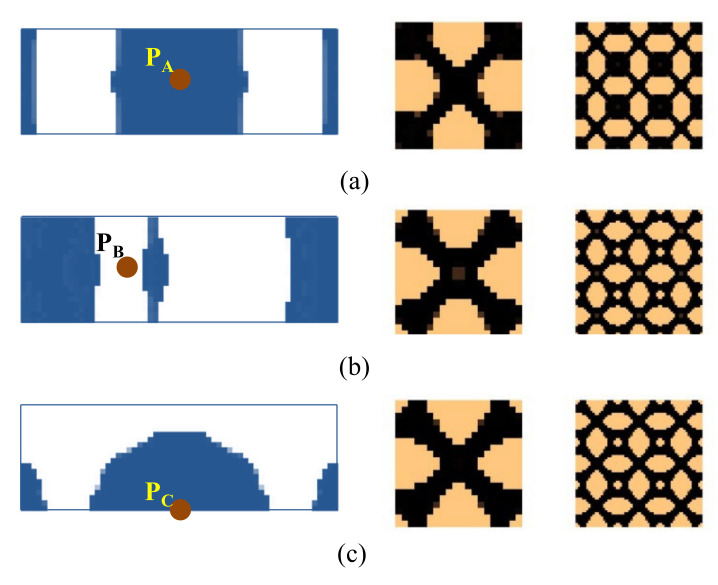
Design results for different excitation positions. (**a**) Excitation position P_A_. (**b**) Excitation position P_B_. (**c**) Excitation position P_C_. The excitation frequency range is 50–100 Hz.

The optimal macrostructures and their associated microstructures are shown in Figure 17. The optimal macrostructures depend on the excitation position. When the excitation position is located at P_A_, the damping material is mainly concentrated in the middle part of the plate. When at P_B_, the damping material is mainly distributed at the two ends of the plate. When at P_C_, the damping material is concentrated around the excitation position. All the optimal solutions in macro-scale are deemed to resist the dominant deformation of the plate in the first bending eigenmode. For the microstructure, all the design results are similar, with only a slight difference at the corner of these microstructures.

**Figure 18:**
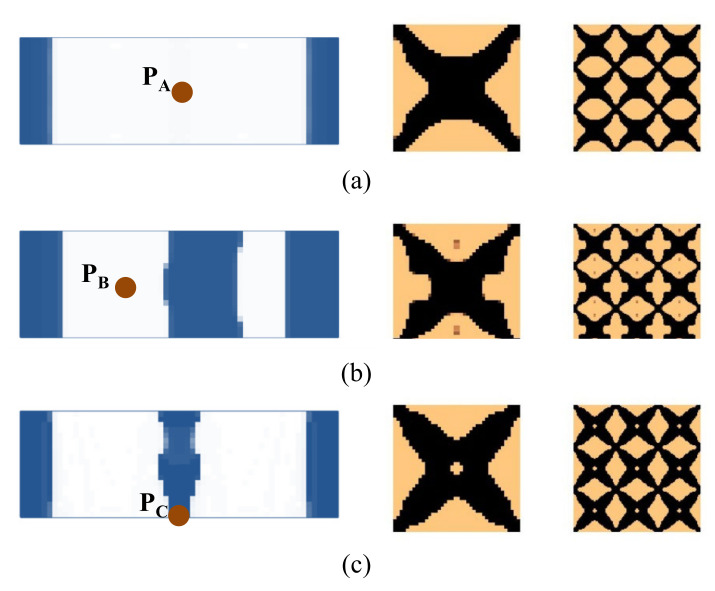
Design results are obtained by varying the excitation positions. (**a**) Excitation position P_A_. (**b**) Excitation position P_B_. (**c**) Excitation position P_C_. The excitation frequency is 300 Hz.

We further consider three test cases under an excitation frequency of 300 Hz. The optimal results are shown in Figure 18. The optimal topologies of the macrostructure depend on the excitation position and frequency. However, the microstructure is similar when the excitation frequencies are the same, except that there are some inconsiderable differences for the reasons of different objectives and load conditions being used.

#### 4.2.3. Composite Plate with Four Corners Fixed

**Figure 19:**
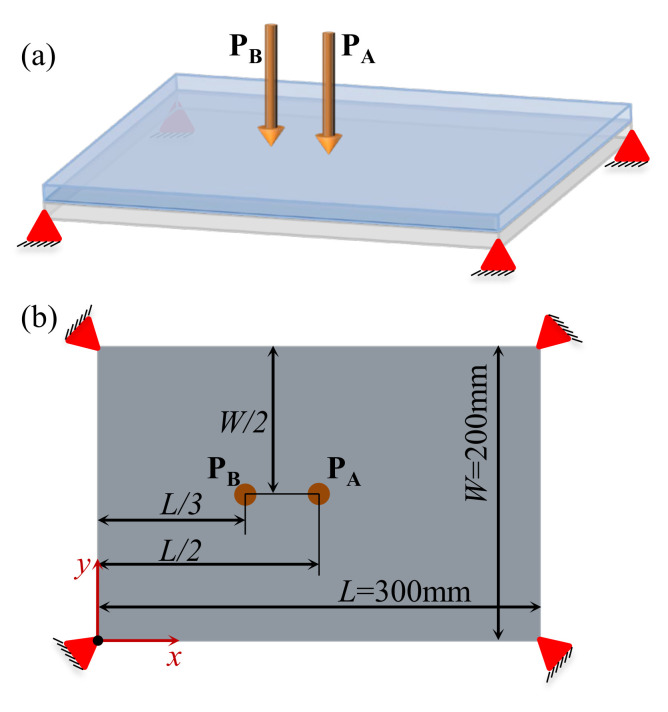
A rectangular plate clamped with four corners. (**a**) Three-dimensional view. (**b**) Top view of the plate.

**Figure 20:**
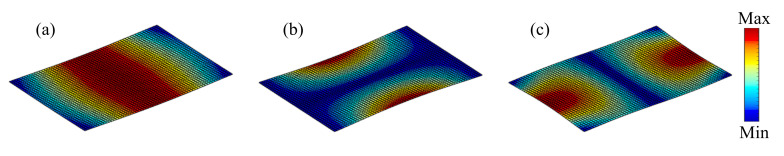
The first three eigenmodes of the plate with four corners fixed. (**a**) The first eigenmode at 64 Hz. (**b**) The second eigenmode at 137 Hz. (**c**) The third eigenmode at 171 Hz.

To further verify the effectiveness of the proposed method, here we consider a composite plate that is fixed at the four corners (Figure 19). The dimension of the plate is 360 × 250 mm. Two different excitation positions are prescribed: P_A_ is located at (*L*/2, *W*/2), and P_B_ is located at (*L*/3, *W*/2). Figure 20 shows the first three eigenmodes with eigenfrequencies of 64, 137, and 171 Hz, respectively. The first eigenmode is bending, the second eigenmode is torsion along the length direction, and the third eigenmode is torsion along the width direction.

**Figure 21:**
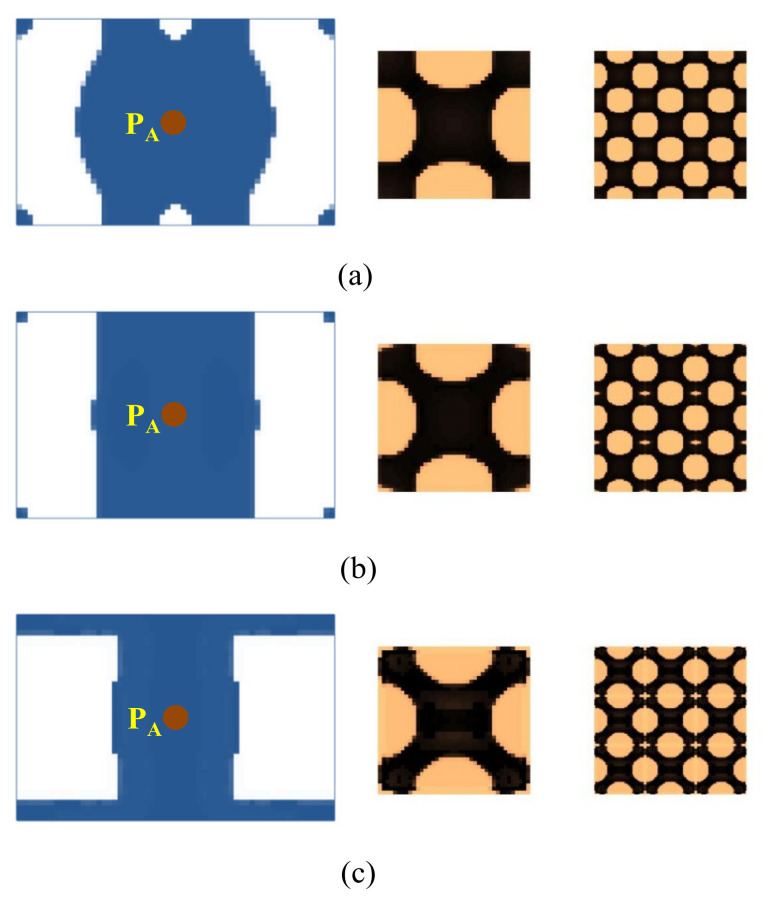
Design results obtained by varying the excitation frequency at the excitation position P_A_. (**a**) Excitation frequency 50 Hz. (**b**) Excitation frequency 100 Hz. (**c**) Excitation frequency 140 Hz.

**Figure 22:**
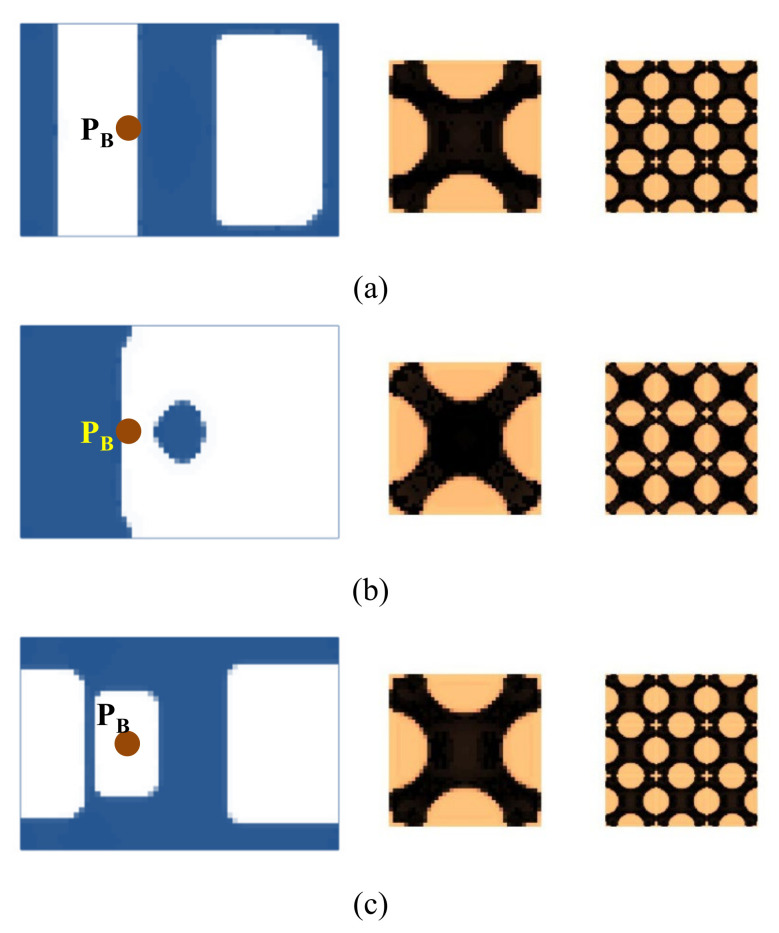
Design results obtained by varying the excitation frequency at excitation position P_B_. (**a**) Excitation frequency 50 Hz. (**b**) Excitation frequency 100 Hz. (**c**) Excitation frequency 140 Hz.

Figure 21 shows the optimal design results with excitation frequencies of 50 Hz, 100 Hz, and 140 Hz at excitation position P_A_. Figure 22 shows the results at excitation position P_B_. All optimal structures have clear binary layouts in both macro- and micro-scales. From the macrostructure topologies, the damping composite is mainly distributed at the center of the plate to resist the deformation around the excitation position P_A_. The material layout of excitation frequency 140 Hz is placed at the center, top and bottom of the plate. This is because the first and second eigenmodes dominate most of the deformation of the structures. When the excitation point is placed at P_B_, the composite material differs from the case where the excitation point is placed at P_A_; they are mainly distributed at the center of the plate to resist the deformation of the plate. For the microstructure topologies, all design cases are similar. The “X” type microstructures are obtained, except for a slight difference at the corner.

**Figure 23:**
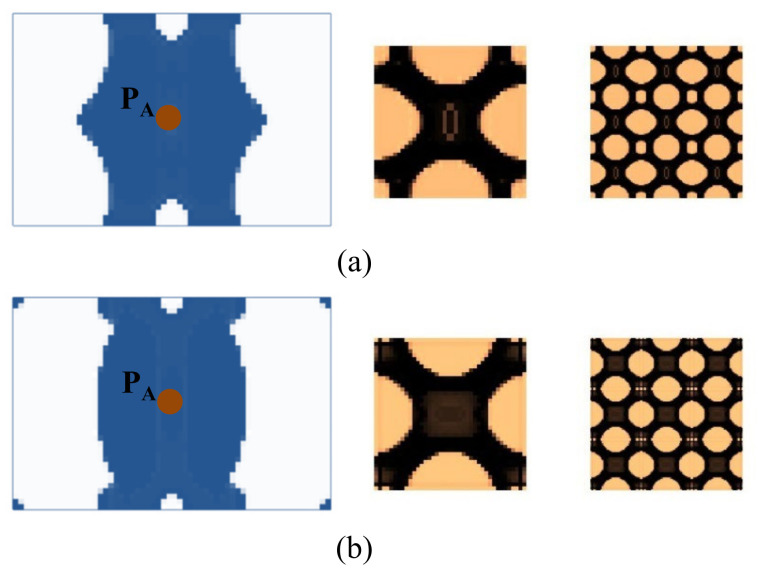
Design results obtained at the same excitation position P_A_ and different excitation frequency ranges. (**a**) Excitation frequency 0–50 Hz. (**b**) Excitation frequency 70–100 Hz.

**Figure 24:**
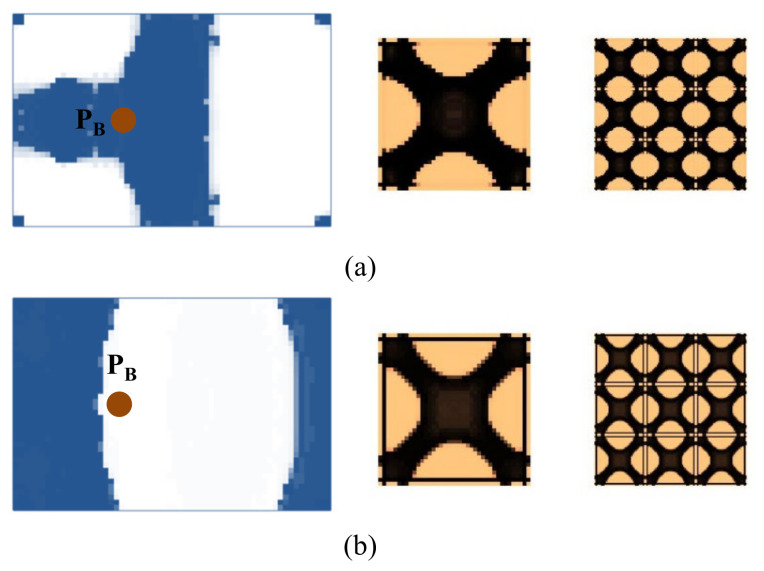
Design results obtained at excitation position P_B_ and different excitation frequency ranges. (**a**) Excitation frequency 0–50 Hz. (**b**) Excitation frequency 70–100 Hz.

We further consider several test cases with two different excitation frequency ranges, which are 0–50 Hz and 70–110 Hz. The optimization results for the case where the excitation point is located at P_A_ and P_B_ are shown in Figure 23 and Figure 24, respectively. When at position P_A_, the optimal macrostructures are shown in Figure 23a,b, which are similar to the results shown in Figure 21a,b. This is because the dominant vibration modes are the same. The optimal microstructures are also similar. For the case where the excitation position is located at P_B_, the macrostructures shown in Figure 24a,b are different from those shown in Figure 22a,b. Notably, the layout shown in Figure 24a is the most efficient one to resist the first bending eigenmode. The microstructures are similar for the excitation frequency in the range of 0–50 Hz but are different in the range of 70–100 Hz.

#### 4.2.4. Composite Plate with Non-Design Domain

**Figure 25:**
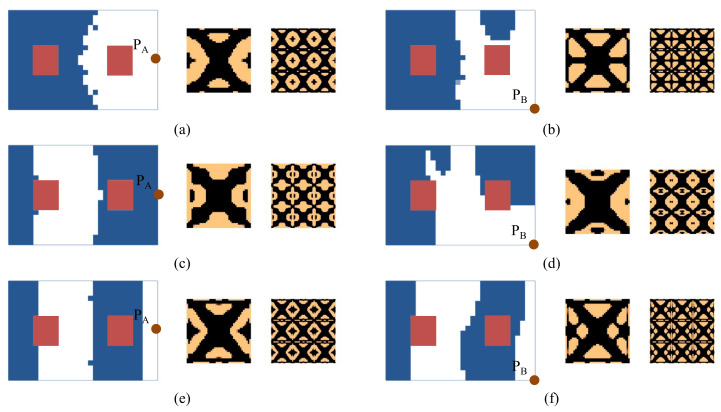
Design results of the cantilever composite plate shown in Figure 5. (**a**) Excitation frequency 0–60 Hz, excitation point P_A_. (**b**) Excitation frequency 0–60 Hz, excitation point P_B_. (**c**) Excitation frequency 90–200 Hz, excitation point P_A_. (**d**) Excitation frequency 90–200 Hz, excitation point P_B_. (**e**) Excitation frequency 300–400 Hz, excitation point P_A_. (**f**) Excitation frequency 300–400 Hz, excitation point P_B_.

**Figure 26:**
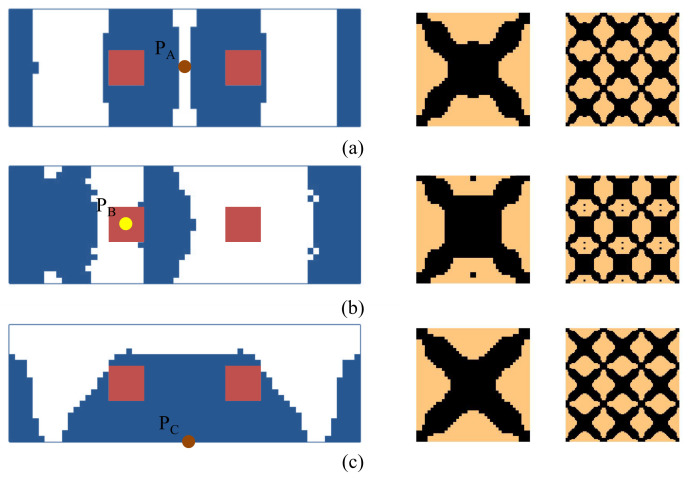
Design results of the composite plate clamped with two opposite edges are shown in Figure 15. (**a**–**c**) correspond to the excitation points P_A_, P_B_, and P_C_, respectively. The excitation frequency is 50–100 Hz.

**Figure 27:**
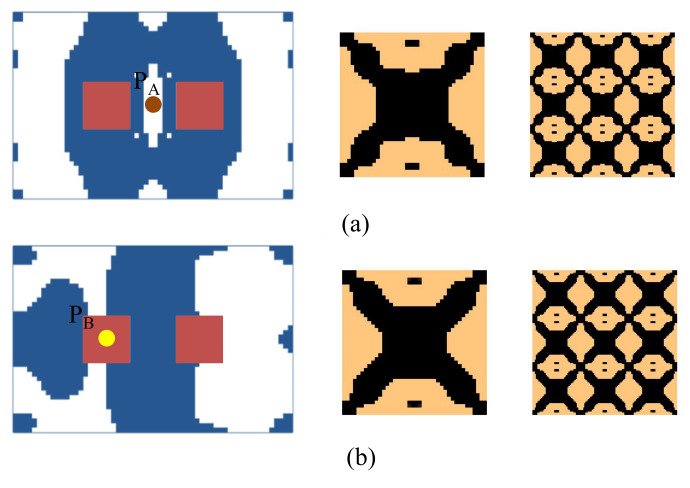
Design results the composite plate with four corners fixed shown in Figure 19. (**a**,**b**) correspond to the excitation points P_A_ and P_B_, respectively. The excitation frequency is 0–50 Hz.

In this section, several design models, including the non-design domain, will be considered. For all the test cases, the initial guess 1 is used. The design model for the first test case is shown in Figure 5, and the design results are shown in Figure 25. In these test cases, on a macro-scale, the blocks in red color are the non-design domains. Comparing the obtained results with those shown in Figure 7, Figure 9 and Figure 10, they have similar macrostructures with the only exception in the non-design domain(s). However, there are some differences in the microstructure. The design model for the second test case is shown in Figure 15, and the design results are shown in Figure 26. The design model for the third test case is shown in Figure 19, and the design results are shown in Figure 27. Similar conclusions can be drawn as the first design case. For the reason of the existence of the non-design domains, the composite material layouts in macro-scale have some differences near the region of the predefined non-design domains.

#### 4.2.5. Composite Plate with Instrument Installed on It

**Figure 28:**
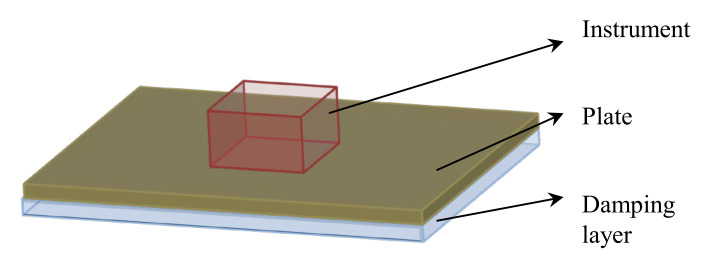
Composite plate with an instrument installed at the center.

Here we present the last design example in this paper, which is shown in Figure 28. The dimension of the plate is 400 × 300 mm. The four sides of the plate are fixed. There is an instrument (100 × 60 mm) being placed at the center of the plate, and its mass is 200 g. We assume that the mass of this instrument is uniformly distributed on the contact area between the instrument and the plate.

**Figure 29:**
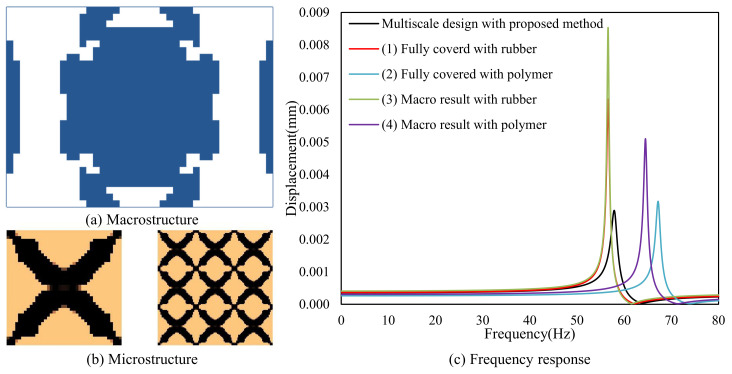
The design result. (**a**) The macrostructure. (**b**) The microstructure and unit cell. (**c**) The frequency response.

The excitation frequency range is 0–50 Hz, and the excitation point is located at the center of the plate. The design results are shown in Figure 29. Four different reference cases are used to compare the performances: (1) fully covered with rubber, (2) fully covered with polymer, (3) macro-scale result with rubber, in which the macrostructure is the same as Figure 29a, and the microstructure is fully covered with rubber, and (4) macro-scale result with polymer, in which the macrostructure is the same as Figure 29a, and the microstructure is fully covered with polymer. From the results, it can be found that, in the specified frequency range of 0–50 Hz, the response of the multi-scale design is lower than the cases of (1) and (3), but a little higher than the cases of (2) and (3). This is because stiffness plays an important role in the low-frequency range. From the results of 0–80 Hz, the multi-scale design result has the minimum response at the first eigenfrequency. This example further demonstrates the effectiveness of the proposed design methodology.

## 5. Conclusions

A concurrent topology optimization method has been proposed to support the design of the composite plates with optimal dynamic performance under harmonic loading. The optimal structures are obtained by performing topology optimization in both macro- and micro-scales simultaneously. In the macroscale, finite element analyses were performed to calculate the structural performance, while in the microscale, the effective material properties were extracted. Based on this two-scale analysis, the microstructure information is integrated into the macrostructure analysis. In turn, the micro-scale sensitivity makes use of the macrostructure displacement field so that the global dynamic compliance is considered in the microscale topology change. The MMA is used to update the design variables, and finally, the optimal multi-scale structures are obtained in both scales.

Numerical examples indicated that the optimal solutions in both scales depend on the loading conditions and boundary conditions. For microscale optimization, the soft rubber damping material in the optimal microstructure unit cell is interconnected. The strong structural connectivity imparts high stiffness and damping to optimal damping composite, which is useful to mitigate undesired vibration. For the macroscale optimization, the excitation frequency range and positions have a significant effect on optimal configurations. The optimal macrostructure has the largest resistance against the deformation of the eigenmodes below and around the excitation frequency. The numerical results demonstrate the effectiveness of the proposed method to obtain efficient macrostructural and microstructural solutions with minimum dynamic compliance. Meanwhile, some general design rules for the design of the composite structures with improved vibration performance can be concluded as follows: (1) at the microscale, the most efficient damping composite architecture should have an interconnected soft phase. In most cases, the “X” type microstructure is efficient and useful for vibration reduction, and (2) at the macroscale, the optimal material layout depends on the eigenmodes. For example, the damping material should be placed at positions such that the resulting structures can resist the deformation of eigenmodes, and at the same time, can improve the structural stiffness. The proposed optimization method can be extended to the design of the constrained-layer damping composite structure or other types of structural components with optimal dynamic performance.

## Figures and Tables

**Table 1:** Material properties of base material and the two different damping materials.

Material	Density (kg/m^3^)	Young’s Modulus (GPa)	Poisson’s Ratio	Loss Factor
Metal (Aluminum alloy)	2700	70	0.29	
Material A (Rubber)	1500	0.05	0.4	1
Material B (Polymer)	1000	2	0.4	0.05

**Table 2:** Comparison between the proposed model and commercial software Altair Hyperworks.

Properties	Fully Covered with Polymer			Fully Covered with Rubber			The Structure in Figure 3		
	Commercial	Proposed	Error	Commercial	Proposed	Error	Commercial	Proposed	Error
The first modal loss factor	0.01342	0.01412	5.21%	0.00973	0.00977	0.42%	0.01189	0.01288	8.38%
The second modal loss factor	0.01238	0.01310	5.83%	0.00870	0.00881	1.30%	0.01063	0.01151	8.26%
The third modal loss factor	0.01320	0.01395	5.68%	0.00955	0.00963	0.85%	0.01169	0.01258	7.60%
The first eigenfrequency (Hz)	265.2	266.8	0.60%	212.8	213.1	0.18%	238.7	237.9	0.35%
The second eigenfrequency (Hz)	796.0	810.5	1.82%	647.4	656.3	1.38%	718.7	730.4	1.62%
The third eigenfrequency (Hz)	1634.6	1654.7	1.23%	1313.9	1324.7	0.82%	1473.2	1479.9	0.46%
CPU time (s)	108	1.03		100	1.04		99	1.16	

## Data Availability

A detailed procedure and flowchart of the proposed method have been presented in Section 3, and one can follow them and reproduce the results. In case of further queries, please contact the corresponding author.
